# High dose and hepatobiliary dysfunction are associated with hand-foot syndrome in patients with lymphoma using pegylated liposomal doxorubicin: a retrospective study

**DOI:** 10.1186/s40360-021-00529-3

**Published:** 2021-10-25

**Authors:** Yanfang Zhao, Wenjia Su, Guohua Liang, Xiaoyu Shan, Weiwei Ma, Dabei Tang, Liru Li, Xingjian Niu, Shu Zhao, Qingyuan Zhang, Wenhui Zhao

**Affiliations:** 1 Department of Medical Oncology, Harbin Medical University Cancer Hospital, Harbin, 150081 China; 2 Department of Lymphoma, The First Hospital Affiliated to Harbin Medical University, Harbin, Heilongjiang 150040 P. R. China

**Keywords:** Pegylated liposomal doxorubicin, Lymphoma, Hand-foot syndrome

## Abstract

**Purpose:**

In clinical practice, the risk factors for pegylated liposomal doxorubicin-related hand-foot syndrome remain unclear. The purpose of this study was to determine the risk factors associated with hand-foot syndrome in patients with lymphoma using pegylated liposomal doxorubicin.

**Methods:**

This retrospective descriptive analysis included patients with lymphoma who received PLD treatment (≥ 2 cycles of chemotherapy) at our cancer centre and had complete follow-up data from January 2016 to February 2020. Clinical, laboratory data, as well as the occurrence of hand-foot syndrome (incidence, location, severity, impact on follow-up chemotherapy) were obtained. The primary end point was the incidence of hand-foot syndrome, which was classified according to the “Common Terminology Criteria for Adverse Events” (Version 4.0). A multivariate logistic regression analysis was used to identify risk factors for hand-foot syndrome in patients with lymphoma using doxorubicin liposomes.

**Findings:**

A total of 167 patients met the inclusion criteria. 58 developed HFS, of which 45 occurred after the second course of chemotherapy. The multivariate logistic regression analysis revealed that a dose increase of pegylated liposomal doxorubicin and hepatobiliary dysfunction were significantly associated with an increased risk for hand-foot syndrome(dose intensity, OR = 6.479; 95% CI, 1.431–29.331 [*P* = 0.015]; history of gallstones, OR = 14.144, 95% CI, 1.512–132.346 [*P* = 0.020]; alanine aminotransferase, OR = 1.194, 95% CI, 1.056–1.350 [*P* = 0.005]; aspartate aminotransferase, OR = 1.162, 95% CI, 1.010–1.336 [*P* = 0.035]; and glutamine transpeptidase, OR = 1.092, 95% CI, 1.016–1.174 [*P* = 0.018]).

**Implications:**

These findings contribute to the risk assessment of patients with lymphoma before using pegylated liposomal doxorubicin. For patients with the above risk factors, preventive measures should be taken in advance to reduce the incidence of HFS.

## Introduction

Anthracycline drugs have significant therapeutic activity in a variety of cancer types [1] and are important in the treatment of lymphoma. However, the effectiveness of these drugs (especially doxorubicin) is limited by substantial toxicity. Doxorubicin can cause cumulative damage to the heart muscle, and most of it is irreversible damage, which limits the repeated use of the drug [2]. In the early 1980s, with the breakthrough of pharmaceutical research, it was found that liposomes can be used as a carrier for anthracycline antibiotics, “buffering” toxicity, while retaining strong antitumour activity, thus improving the therapeutic index of anthracycline antibiotics [3]. Pegylated liposomal doxorubicin (PLD) is doxorubicin encapsulated in liposomes, which incorporate phospholipid conjugates of methoxy-polyethylene glycol [4]. PLD is used as a drug for the treatment of various malignancies, including AIDS-related Kaposi’s sarcoma, ovarian cancer, lymphoma, metastatic breast cancer, and multiple myeloma [5]. Compared with conventional anthracycline drugs, the circulation time of PLD is long, its payload remains stable, and its accumulation in tumours with high vascular permeability has important advantages. The ability of PLD to reduce many of the adverse side effects of doxorubicin (including reducing cardiotoxicity) is now clear, but the subsequent emergence of special adverse reactions (such as hypersensitivity, mucositis, and hand-foot syndrome) has undoubtedly added to clinicians’ concerns about the use of PLD.

Hand-foot syndrome (HFS), also known as palmar-plantar erythrodysesthesia (PPE) or Burgdorf disease, is a skin side effect of a series of chemotherapy drugs [6, 7]. Symptoms of mild HFS are usually described as numbness or paresthesia and oedema. Continued medication may cause more severe symptoms, such as erythema, blisters, chapped skin, erosions and ulcers. As its name implies, HFS lesions mainly occur on the palms and soles, but other skin areas of the body may also become affected, especially under the breasts and in the armpits, groin, or abdominal area. Hackbarth found that 80% of patients treated with PLD develop mild HFS [8].

According to reports, many chemotherapy drugs are associated with HFS [9]. For any grade of adverse reactions, the incidence of PLD-related HFS is up to 50%, and for the occurrence of grade 3 or more adverse reactions, the incidence is approximately 20% [10]. Although the incidence of HFS in lymphoma patients is not as high as that in other cancer types, it greatly reduces the patient’s compliance with medication and affects the patient’s quality of life. The initial presentation of HFS is numbness, redness, and pain in the hands and feet, but in severe cases of HFS, patients find it difficult to walk and lose the ability to hold objects, which seriously damages their daily life and may affect subsequent cancer treatment. Therefore, the prevention and treatment of HFS are crucial. However, the risk factors for HFS in patients with lymphoma using PLD are not yet clear, and related data are still scarce.

With the gradual deepening of the research on the mechanism of HFS, it is possible to clinically explore the related factors of its occurrence. To use PLD more selectively and reasonably in the future, we conducted a retrospective study on the data of lymphoma patients admitted to our hospital in recent years who received chemotherapy with a PLD regimen to analyse the related factors of HFS caused by PLD.

## Patients and methods

### Research objects

This study included all patients who were newly diagnosed with lymphoma by pathology and received ≥2 courses of chemotherapy, including PLD, in the Cancer Hospital of Harbin Medical University between January 2016 and February 2020. We excluded patients with incomplete clinical data, those who were lost follow-up, and those who had primary cutaneous lymphoma, cutaneous T-cell lymphoma, and other skin lesions.

### Data collections

We collected relevant clinical and laboratory data from all patients. The clinical data were as follows: sex, age, body mass index (BMI), clinical stage, ECOG score, IPI score, presence or absence of large tumour mass, pathological type, group B symptoms, lactate dehydrogenase (LDH), history of viral hepatitis(including inactive carrier of HBV), and history of gallstones. The laboratory data were as follows: alanine aminotransferase (ALT), aspartate aminotransferase (AST), glutamine transpeptidase (GGT), total bilirubin (TBIL), and monocyte count (MONO) at baseline. At the same time, the severity of HFS, the course of treatment with PLD, dose adjustment information.

### Diagnosis and classification of HFS

We use The National Cancer Institute’s Common Terminology Criteria for Adverse Events (NCI-CTCAE, v4.0) classification system for skin toxicity and the World Health Organization classification to classify HFS [11, 12].

### Dose adjustment

If the patient has grade 1 HFS, there is no need to adjust the PLD dose, so the patient should continue taking the original dose. If grade 2 HFS occurs, the patient should postpone the use of PLD for 2 weeks or until it returns to grade 0–1; if the recovery does not resume after 2 weeks, the use of PLD should be suspended. When grade 3 HFS occurs, the patient should postpone the use of PLD for 2 weeks or until it returns to grade 0–1 and 75% of the original dose should be administered with an unchanged administration interval; if HFS does not return to grade 0–1 after 2 weeks, the use of PLD should be suspended [13].

### Statistical methods

SPSS 25.0 statistical software (SPSS Inc., Chicago, USA) was used to analyse the collected possibly related factors. The measurement data are expressed as the mean ± standard deviation ($\overline{x}$±s), and comparisons between groups were performed by independent sample t-tests. The classification data are expressed as a percentage (%), and the difference between the two samples was analysed using the chi-square test. Multivariate binary logistic regression analysis was performed on the influencing factors with statistically significant differences screened by single factor analysis. *P* < 0.05 was considered statistically significant.

## Results

### Chemotherapy regimens

The chemotherapy regimens of 167 patients with lymphoma who used PLD are shown in Table 1.

**Table 1:** Chemotherapy regimens for lymphoma

Types of lymphoma	Chemotherapy regimens	Number of patients	Chemotherapy cycle (days)/time	Dose intensity of PLD(mg/m^2^)
NHL	CDOP	19	21/d1	30
		30		40
	R-CDOP	31	21/d2	30
		42		40
	CDOPE	3	21/d1	30
		7		40
	R-CDOPE	3	21/d2	30
		4		40
	PDD	1	21/d1	40
	VRCDOP	2	21/d2	30
		1		40
HL	ABVD	24	28/d1,d15	40/d1and d15

*Abbreviations: *NHL* Non-Hodgkin’s lymphoma, *HL* Hodgkin’s lymphoma, *R-CDOP* (rituximab, cyclophosphamide, PLD, vincristine, prednisone), *CDOP* (cyclophosphamide, PLD, vincristine, prednisone), *CDOPE* (cyclophosphamide, PLD, vincristine, prednisone, etoposide), *PDD* (bortezomib, PLD, dexamethasone), *VRCDOP* (bortezomib, rituximab, cyclophosphamide, PLD, vincristine, prednisone), *ABVD* (PLD, bleomycin, vinblastine, dacarbazine)

Before intravenous PLD, the patients were given dexamethasone 10 mg intravenously or oral prednisone according to the chemotherapy regimen to prevent allergic reactions.

The study group consisted of 167 patients with lymphoma who received PLD treatment (≥ 2 cycles of chemotherapy). NHL accounted for 85.6% (143/167), HL accounted for 14.4% (24/167), NHL accounted for 76.6% (128/167) of B-cell lymphoma, and T-cell lymphoma accounted for 9.0% (24/167). All pathological types are summarized in detail in Table 2.

**Table 2:** Pathological types of lymphoma

Pathological type		Number of cases	Total ratio
NHL	DLBCL	103(61.6%)	76.6%
	FL	8(4.8%)	
	MCL	10(6.0%)	
	MZL	7(4.2%)	
	PTCL	11(6.6%)	9.0%
	AITL	3(1.8%)	
	ALCL	1(0.6%)	
HL		24(14.4%)	14.4%

*Abbreviations: *DLBCL* diffuse large B-cell lymphoma, *FL* follicular lymphoma, *MCL* mantle cell lymphoma, *MZL* marginal zone lymphoma, *PTCL* peripheral T cell lymphoma, *AITL* angioimmunoblastic T-cell lymphoma, *ALCL* anaplastic large cell lymphoma

A total of 167 patients with lymphoma who were treated with a regimen including PLD were enrolled, and 58 patients had HFS, with an incidence rate of 34.7%, including 36 cases of grade 1 HFS, accounting for 21.5%; 18 cases of grade 2 HFS, accounting for 10.8%; and only 4 cases of grade 3 and above HFS, accounting for 2.4%. In 45 of these patients, HFS occurred after the second course of chemotherapy, accounting for 77.6%. The detailed clinical characteristics are shown in Table 3.

**Table 3:** Clinical characteristics of patients treated with PLD for lymphoma

Clinical characteristics		Number of cases (Percentage)
Gender	Male	72(43.1%)
	Female	95(56.9%)
Age	≤60	73(43.7%)
	> 60	94(56.3%)
Clinical stage	I-II	77(46.1%)
	III-IV	90(53.9%)
ECOG score	< 2	108(64.7%)
	≥2	59(35.3%)
IPI score	1–2	94(56.3%)
	3–5	73(43.7%)
Whether there are large tumor masses	Yes	28(16.8%)
	No	139(83.2%)
Pathological type	HL	24(14.4%)
	NHL	143(85.6%)
Group B symptoms	Yes	64(38.3%)
	No	103(61.7%)
LDH	Normal	65(38.9%)
	Elevated	102(61.1%)
History of gallstones	Yes	11(6.6%)
	No	156(93.4%)
Viral hepatitis	Yes	23(13.8%)
	No	144(86.2%)

*Abbreviations: *IPI* International Prognostic Index, *LDH* lactate dehydrogenase

### Analysis of related risk factors

Statistical analyses of the clinical and laboratory data of 167 patients showed the following. (1) For dose intensity [40 mg·m^2^: 43.8% (46/105) vs 30 mg·m^2^: 19.3% (12/62), *P* = 0.001], history of gallstones (*P* = 0.025), ALT (30.28 ± 15.93 U/L vs 14.14 ± 4.91 U/L, *P* = 0.000), AST (29.17 ± 8.79 U/L vs 19.27 ± 4.29 U/L, *P* = 0.000), and GGT (46.24 ± 35.60 U/L vs 19.23 ± 7.38 U/L, *P* = 0.000), the differences were not statistically significant. (2) Regarding sex differences in HFS patients, the incidence of HFS in females was 38.9%, and the average incidence of HFS in males was 29.1%; thus, the incidence in females was greater than that in males, but the difference was not significant. (3) The average age of patients with HFS was 59 ± 9.76 years, and the average age of patients without HFS was 61 ± 14.60 years. The average age of patients with HFS was higher than that of patients without HFS. (4) The incidence of HFS in patients with group B symptoms was 39.0%, and the incidence of HFS in patients without group B symptoms was 32.0%. The incidence of HFS in patients with group B symptoms was higher than that of patients without group B symptoms, but the difference was not statistically significant. (5) The incidence of HFS in patients with stage I-II disease was 41.5%, and the incidence of HFS in patients with stage III-IV disease was 29.0%. Patients with early stage disease were more likely to develop HFS than patients with advanced stage disease, but there was no significant difference between these two groups. (6) The incidence of HFS in patients with HL was 45.8%, and the incidence of HFS in patients with NHL was 32.8%. HL patients were more prone to HFS than NHL patients, but this difference was not statistically significant. (7) The incidence of HFS in patients with a history of viral hepatitis was 52.2%, and the incidence of HFS in patients without a history of viral hepatitis was 31.9%. The incidence of HFS in patients with a history of viral hepatitis was higher than that of patients without a history of viral hepatitis, but the difference was not statistically significant. (8) The average number of treatments in patients with HFS was 5.47 ± 2.00, and the average number of treatments in patients without HFS was 5.36 ± 1.52; there was no significant difference between them. (9) For baseline TBIL (13.62 ± 5.04 μmol/L vs 13.51 ± 4.37 μmol/L, *P* = 0.889) and baseline MONO (0.41 ± 0.12 10^9^/L vs 0.42 ± 0.46 10^9^/L, *P* = 0.860), the results were not statistically significant. Table 4 summarizes the relationships between HFS and the possible related factors.

**Table 4:** Comparison of clinical and laboratory data between patients with HFS and those without HFS

Factor		HFS (*n* = 58)	Non-HFS (*n* = 109)	*P* value
Sex	Male	21(36.2%)	51(46.8%)	0.189
	Female	37(63.8%)	58(53.2%)	
Age		59 ± 9.76	61 ± 14.60	0.352
BMI(kg/m^2^)		23.93 ± 2.17	23.34 ± 2.32	0.114
Dose intensity (mg·m^2^)	30	12(20.7%)	50(45.9%)	0.001*
	40	46(79.3%)	59(54.1%)	
Group B symptoms	Yes	25(43.1%)	39(35.8%)	0.354
	No	33(56.9%)	70(64.2%)	
Clinical stage	I-II	32(55.2%)	45(41.3%)	0.086
	III-IV	26(44.8%)	64(58.7%)	
Presence of large tumour masses	Yes	7(12.1%)	21(19.3%)	0.236
	No	51(87.9%)	88(80.7%)	
Pathological type	HL	11(19.0%)	13(11.9%)	0.248
	NHL	47(81.0%)	96(88.1%)	
History of gallstones	Yes	8(13.8%)	3(2.8%)	0.006*
	No	50(86.2%)	106(97.2%)	
Hepatitis	Yes	12(20.7%)	11(10.1%)	0.058
	No	46(79.3%)	98(89.9%)	
Treatment cycle		5.47 ± 2.00	5.36 ± 1.52	0.721
Curative effect	CR + PR	47(81.0%)	97(89.0%)	0.155
	SD + PD	11(19.0%)	12(11.0%)	
Baseline ALT		30.28 ± 15.93	14.14 ± 4.91	< 0.001*
Baseline AST		29.17 ± 8.79	19.27 ± 4.29	< 0.001*
Baseline GGT		46.24 ± 35.60	19.23 ± 7.38	< 0.001*
Baseline TBIL		13.62 ± 5.04	13.51 ± 4.37	0.889
Baseline MONO		0.41 ± 0.12	0.42 ± 0.46	0.860

*Abbreviations:*ALT* alanine aminotransferase, *AST* aspartate aminotransferase, *GGT* glutamine transpeptidase, *TBIL* total bilirubin, *MONO* monocyte count

Multivariate logistic regression analysis was conducted on the above relevant factors with significant differences. The results showed that dose intensity (40 mg·m^2^), history of gallstones, ALT elevation, AST elevation, and GGT elevation were independent risk factors for HFS (Table 5).

**Table 5:** Multivariate logistic regression analysis of risk factors

Influence factor	B	Standard error	Wald	*P* value	Exp(B)	95% CI
Dose intensity	1.869	0.770	5.882	0.015	6.479	1.431 ~ 29.331
History of gallstones	2.649	1.141	5.392	0.020	14.144	1.512 ~ 132.346
ALT	0.177	0.063	7.961	0.005	1.194	1.056 ~ 1.350
AST	0.150	0.071	4.423	0.035	1.162	1.010 ~ 1.336
GGT	0.088	0.037	5.645	0.018	1.092	1.016 ~ 1.174

*Abbreviations: Dose intensity, Dose per cycle

## Discussion

HFS is a well-documented and relatively common skin reaction that is associated with multiple chemotherapy drugs. Capecitabine, 5-fluorouracil, cytarabine and PLD are the most common drugs that cause HFS [14]. HFS has a variety of symptoms, ranging from mild discomfort to palm and foot pain, which may limit function and hinder the patient’s daily activities, but usually disappears within 1 to 5 weeks after stopping the drug [14]. Although HFS is not life-threatening, it may be the main reason for decreased patient compliance and may have a serious impact on quality of life. In addition, with the expansion of the application of PLD in lymphoma, the incidence of HFS may increase. Therefore, the prevention and treatment of these reactions are essential to improve the quality of life of cancer patients and avoid unnecessary dose adjustments that may affect the therapeutic effect.

The mechanism of PLD causing HFS may be related to the strong cytotoxicity inherent to doxorubicin, the long half-life of PLD in the blood, or the interaction between doxorubicin and a large number of Cu(II) ions in skin tissue to produce reactive oxygen species [15]. Due to the rich distribution of capillaries, which are composed of a single layer of endothelial cells, in the fingertips and toes and high blood flow, the drug penetrates from the capillary wall to the interstitial space immediately after slight stimulation; the hands and feet are rich in sweat glands, and the drugs are more permeable to sweat in the stratum corneum [16, 17]; moreover, keratinocytes, blood cells and fibroblasts will produce inflammatory cytokines, resulting in vasodilation, increased vascular permeability, redness, fever, and swelling [15]. Eventually, the cumulative effect increases with the toxicity of the chemotherapy cycle, further forming the described HFS lesions. HFS can be alleviated by adjusting the dose or dosing intervals, using cooling methods, wearing loose clothing, and using emollient chemicals. Severe HFS may require delayed chemotherapy.

The early identification of mild symptoms, the improvement of patients’ relevant education and the close follow-up of doctors are key elements of HFS prevention management. In a nursing support plan, including instructions for providing information on the potential toxicity of PLD and preventing and treating PLD toxicity led to a very low incidence of severe HFS, not exceeding 4% of patients receiving treatment [18]. The current effective way to control HFS is to modify the treatment method, such as prolonging the dosage interval, reducing the dose or interrupting the use of PLD. The above measures can improve the symptoms of HFS within 1–2 weeks, and for level 1 reactions, supportive treatments and emollient agents can be used. To date, no large-scale controlled study has evaluated the therapeutic effect and preventive measures of HFS.

Regarding the preclinical and clinical pharmacokinetics of PLD, the clearance rate of PLD is easily saturated at higher doses. Related studies have reported that the clearance rate of the standard dose of 40–60 mg/m^2^ (the dose used by patients with solid tumours) is much slower than that of the lower dose 20 mg/m^2^ (such as the dose used in Kaposi’s sarcoma) [19]. The incidence of HFS is linked to the dose intensity of PLD. When PLD is used at the standard dose of 40 mg·m^2^, the incidence of HFS is 43.8% (46/105), but when the dose intensity of PLD is 30 mg·m^2^, the incidence of HFS is only 19.3% (12/62); the results are statistically significant (*P* = 0.001). Considering the dose dependence, the PLD dose should be individualized.

Animal studies have shown that some doxorubicin is excreted in the urine, but bile is the principal route of doxorubicin excretion after PLD [20]. Consequently, when the patient’s liver function is abnormal or when bile excretion is restricted, PLD may accumulate in the body for a long time, thereby causing HFS. This study (Table 4) showed that the occurrence of HFS is related to a history of gallstones (*P* = 0.025), increased ALT (*P* = 0.000), increased AST (*P* = 0.000), and increased GGT (*P* = 0.000), which are independent risk factors for HFS (Table 5). Hence, in the clinical application of PLD, it is necessary to ask the patient in detail whether he/she has a history of gallstones, to evaluate liver function and other related indicators (ALT, AST, GGT), and then to understand whether the patient has abnormal liver function or abnormal bile excretion. This information provides a reference for avoiding the occurrence of HFS and adjusting the dose of PLD reasonably.

The study found that among the 167 patients with lymphoma who used PLD, 58 patients had HFS, with an incidence of 34.7%. Most of them tolerated PLD well, and most patients with HFS had grade 1–2 HFS, only 4 patients had grade 3 HFS (2.4%), 5 patients postponed the chemotherapy cycle due to HFS of grade 2 or higher, 3 patients adjusted the dose to 75% of the initial dose, and only 1 patient switched to other drugs because of HFS; the HFS of the abovementioned patients recovered well through active symptomatic treatment measures. For any level of response, the incidence of HFS associated with PLD is up to 50%, and for the occurrence of adverse reactions above grade 3, the incidence is approximately 20% [10]. Compared with other tumours, the use of PLD causing HFS in lymphoma is relatively rare, and the severity is greatly reduced. The results of this study (Table 4) show that HL patients are more prone to HFS than NHL patients. Regardless of the lack of a significant difference, it is worthy of further research. Therefore, apart from being related to different tumour types, it may also be because the chemotherapy regimen of NHL patients contains high-dose corticosteroids to avoid allergic reactions on the one hand and prevent and treat PLD-related HFS on the other hand [21].

Compared with conventional preparations, the pharmacokinetic differences among patients treated with PLD are significantly higher [22]. Age, sex, and monocyte count before the cycle seem to be related to the patient’s PLD clearance. The PLD clearance rate of young patients (< 60 years old) is approximately twice as fast as that of elderly patients (≥60 years old), and the clearance rate of PLD among male patients is faster than that of female patients [23, 24]. At the same time, the PLD clearance rate decreased significantly with increasing chemotherapy cycles [25], which is related to the decline in the function of the liver’s mononuclear phagocyte system (MPS) [23]. The greater reduction in PLD clearance is also associated with a decrease in monocyte count before the PLD treatment cycle, which indicates that the toxicity of doxorubicin to the MPS (as evidenced by a decrease in peripheral blood monocyte count) reduces the clearance of PLD via the MPS. The reduction in PLD clearance is clinically significant, as a longer PLD half-life has been assessed and found to be associated with a greater risk of skin toxicity [26]. To improve the response of PLD treatment and minimize toxicity, it is necessary to determine the factors related to variability within and between patients using PLD.

In this study, the data summarized in Table 4 reveal that the age of patients with HFS was older than that of patients without HFS, but this difference did not reach statistical significance, and it was inconsistent with the abovementioned applicable theoretical research results. This may be because this study is a retrospective study and not randomized according to age. Most young people with good physical strength are in the group with high drug dose intensity, so they are greatly affected by the dose intensity factor, but the effect of dose intensity on the occurrence of HFS in older patients was also confirmed. Although the incidence rate in women was greater than that in men, the difference was not statistically significant, and the study sample size needs to be further expanded. Unfortunately, we cannot determine the relationship between BMI, monocyte count and the occurrence of HFS from these data.

In summary, according to the results of this study, factors such as high-dose PLD, history of gallstones and abnormal liver function(ALT, AST, GGT) should be considered in the application of PLD in order to identify patients who are at the main risk of developing its toxicity.

The present study has several limitations. First, the chemotherapy regimens of the patients in this study were not completely consistent, and the interaction between drugs cannot be ruled out, which may have an impact on the results. Second, this is a retrospective study, and the patients could not be reasonably grouped and stratified; moreover, there may be interference between various factors. These factors should be improved in future research. Notwithstanding its limitations, this study provides references for future large-scale prospective, randomized clinical studies and pharmacokinetic studies. Combining these related factors, further research on the association between PLD pharmacokinetics and clinical outcomes (efficacy and toxicity) as well as subsequent large-scale prospective, randomized clinical studies will lead to the development of strategies to optimize PLD treatment.

## Conclusions

In this study, the dose intensity, history of gallstones, ALT, AST, and GGT were significantly different between groups (*P* < 0.05). Among them, increased dose, history of gallstones, ALT elevation, AST elevation, and GGT elevation were independent risk factors for HFS in patients with PLD-treated lymphoma (*P <* 0.05).

## Data Availability

The datasets used and/or analysed during the current study are available from the corresponding author on reasonable request.
